# Supplementary material for: Global hypo-methylation in a proportion of glioblastoma enriched for an astrocytic signature is associated with increased invasion and altered immune landscape
Source: eLife. 2022 Nov 22;11:e77335. doi: 10.7554/eLife.77335 (PMC9681209; doi:10.7554/eLife.77335)
Supplement: Figure 2—source data 1. [file elife-77335-fig2-data1.zip › Figure_2_source_data_1/Figure_2I_J/homerResults/motif13.similar.html]

motif13

## Information for motif13

A
G
T
C
G
T
A
C
G
T
A
C
G
T
A
C
C
G
T
A
A
C
T
G
C
A
T
G
T
C
A
G
A
T
G
C
G
A
C
T
  
Reverse Opposite:  

C
T
G
A
T
A
C
G
A
G
T
C
G
T
A
C
T
G
A
C
G
C
A
T
C
A
T
G
C
A
T
G
C
A
T
G
T
C
A
G
  

|  |  |
| --- | --- |
| p-value: | 1e-40 |
| log p-value: | -9.327e+01 |
| Information Content per bp: | 1.672 |
| Number of Target Sequences with motif | 46.0 |
| Percentage of Target Sequences with motif | 6.34% |
| Number of Background Sequences with motif | 0.4 |
| Percentage of Background Sequences with motif | 0.15% |
| Average Position of motif in Targets | 106.8 +/- 55.5bp |
| Average Position of motif in Background | 9.0 +/- 0.0bp |
| Strand Bias (log2 ratio + to - strand density) | 0.8 |
| Multiplicity (# of sites on avg that occur together) | 1.15 |
| Motif File: | file (matrix) reverse opposite |

### Similar de novo motifs found

|  |  |  |  |  |  |  |  |
| --- | --- | --- | --- | --- | --- | --- | --- |
| Rank | Match Score | Redundant Motif | P-value | log P-value | % of Targets | % of Background | Motif file |
| 1 | 0.603 | A T C G T G A C T A G C T G C A C T A G C A T G C T A G T C A G T G A C T G C A | 1e-39 | -91.402888 | 15.01% | 3.42% | motif file (matrix) |
| 2 | 0.756 | A T G C G A C T A T G C T G A C C G T A A C T G C T A G T A C G T A G C C G T A T A C G T C G A | 1e-36 | -84.508789 | 5.92% | 0.57% | motif file (matrix) |
| 3 | 0.601 | A G C T A G T C G C T A A C T G C T A G T A C G T A G C G A T C G C A T A C T G | 1e-26 | -60.985666 | 7.30% | 1.31% | motif file (matrix) |
| 4 | 0.666 | A T G C G A T C G A T C G T A C G C A T C T A G T A C G A G C T | 1e-24 | -57.020487 | 7.02% | 1.38% | motif file (matrix) |
| 5 | 0.612 | T C A G T A G C G T C A A T G C G C A T A C G T C A T G A C T G | 1e-23 | -55.068859 | 6.89% | 1.08% | motif file (matrix) |
| 6 | 0.606 | C T A G C T A G C T A G C T G A T A C G C T G A T A G C T C G A A T G C G A T C C G A T A T C G C A T G A T C G T A G C | 1e-19 | -44.394329 | 5.10% | 0.72% | motif file (matrix) |
| 7 | 0.648 | A G T C A G T C C G T A A G T C C G T A A C T G A C T G A C T G A G T C A G T C A G T C A G T C C G T A A C T G A G T C | 1e-6 | -16.019182 | 2.89% | 1.05% | motif file (matrix) |
